# Balancing Risk and Reward: Dopamine’s Central Role in Economic Decision-Making

**DOI:** 10.3390/brainsci15080857

**Published:** 2025-08-12

**Authors:** Luca Aquili, Lee Wei Lim

**Affiliations:** 1 School of Management, Ritsumeikan Asia Pacific University, Beppu 874-0011, Japan; 2 DrLim Neuromodulation Lab, Department of Biosciences and Bioinformatics, School of Science, Xi’an Jiaotong-Liverpool University, Suzhou 215123, China; leewei.lim@xjtlu.edu.cn

**Keywords:** financial decision-making, dopamine, risk, reward, reinforcement learning

## Abstract

Dopamine has been increasingly implicated in shaping economic and financial decision-making, yet much of the evidence remains fragmented across paradigms and mechanistic levels, and heavily based on preclinical or clinical populations. This review synthesises pharmacological, neuroimaging, and genetic findings from studies involving healthy human participants, highlighting dopamine’s role in risk-taking, delay discounting, social fairness, reward sensitivity, and feedback learning. It distinguishes between transient state-related effects and stable trait-level influences, and clarifies how dopaminergic tone, receptor subtype activity—particularly D2—and corticostriatal circuitry modulate economic choices. In doing so, the review advances a mechanism-focused framework for understanding adaptive and biased decision strategies.

## 1. Introduction

The catecholamine dopamine has long been investigated for its role in reward-prediction error and reinforcement learning [1,2], executive function [3], working memory [4], and various clinical disorders, including addiction, schizophrenia, and Parkinson’s disease [5,6,7]. At the same time, a growing body of research demonstrates that dopamine modulates economic decision-making, particularly in relation to risk evaluation, delay of gratification, reward sensitivity, and strategic behaviour [8,9,10,11,12]. However, much of this evidence remains under-synthesised, fragmented across paradigms and methodologies, lacking a coherent, mechanistic account of how dopaminergic systems shape financial decision-making.

The present review aims to bridge this gap by providing a comprehensive and up-to-date synthesis of dopamine’s role in economic behaviour, with a particular focus on healthy human participants. To organise this review, we adopt a mechanism-centric structure to foreground the neurobiological processes through which dopamine influences economic choices. Specifically, we examine five interrelated dopaminergic mechanisms:

- Dopamine release and pharmacological modulation
- Receptor subtype contributions (D1, D2, D3, D4)
- Neural circuitry and regional activity, as revealed through neuroimaging
- Genetic polymorphisms affecting dopamine signalling
- Stable trait-level influences, such as investment experience and personality

While the volume and strength of evidence vary across these mechanisms—reflecting how unevenly each has been studied in economic contexts—we include them as conceptually distinct but complementary perspectives on how dopamine contributes to financial decision-making.

Although the review is organised around mechanistic domains, constructs such as impulsivity and delay discounting cut across these categories and are central to intertemporal and risk-based economic choices. Dopaminergic influences on delay discounting have been demonstrated in several studies [13,14,15] and underscore the broader role of dopamine in affecting temporal preferences relevant to economic valuation.

For each mechanism, we survey findings from pharmacological interventions (e.g., L-DOPA, cabergoline, haloperidol), neuroimaging techniques (e.g., fMRI, PET), and genetic association studies (e.g., dopamine D2 receptor gene DRD2, dopamine D4 receptor gene DRD4, dopamine transporter gene DAT1, and catechol-O-methyltransferase COMT). We highlight how these biological factors influence financial behaviours such as risk-taking, delay discounting, belief updating, sensitivity to fairness, and reward valuation. While our primary focus is on genes directly involved in dopaminergic signalling, we acknowledge that other genes—such as TH (tyrosine hydroxylase) and SLC18A2 (VMAT2), as well as more distally linked genes to dopaminergic function such as PPP1R1B (DARPP-32), GRM3 (metabotropic glutamate receptor 3), and BDNF (brain-derived neurotrophic factor)—have been implicated in domains such as impulsivity, cognitive control, and motivation. These traits are relevant to economic decision-making but given the limited direct evidence connecting these genes to financial behaviour, we do not review them in detail here.

A similar rationale applies to norepinephrine, which shares biosynthetic pathways with dopamine and has also been associated with responses to financial loss and uncertainty [16,17]. While clearly relevant, these findings are beyond the mechanistic focus of this review.

We conclude this review with an integrative synthesis that identifies cross-cutting patterns, theoretical implications, and directions for future research. To aid synthesis and cross-comparison, a summary table is provided at the end of each mechanistic Section 2.1, Section 2.2, Section 2.3, Section 2.4 and Section 2.5, organising the reviewed studies by methodological approach, dopaminergic target, economic paradigm, and key findings.

## 2. Dopaminergic Mechanisms in Economic Decision-Making

### 2.1. Dopamine Release and Pharmacological Modulation

This section examines studies that manipulate or measure dopamine availability directly—either through pharmacological interventions (e.g., L-DOPA, tolcapone), invasive recordings, or PET-based assessments of synthesis capacity. Unlike receptor subtype or genetic studies, which infer downstream effects, this literature directly indexes fluctuations in dopamine levels or turnover. These studies cover a range of economic paradigms, from fairness and social decision-making to cognitive control and belief updating. While diverse in task design, they converge on the notion that dopaminergic tone dynamically shapes valuation, behavioural flexibility, and context-sensitive adaptations.

Batten et al. [18] used deep brain stimulation and electrochemical recordings in Parkinson’s disease patients to compare dopaminergic activity in the substantia nigra when rejecting unfair offers from human versus computer partners in an Ultimatum Game. Rejections of human offers were associated with greater dopamine release, suggesting that social context modulates dopaminergic responses to norm violations. This finding highlights dopamine’s role beyond mere reward prediction, implicating it in socially salient decision-making. A complementary result comes from a Dictator Game study [19], where administration of tolcapone, a COMT inhibitor that elevates prefrontal dopamine—led to stronger behavioural responses to unfair offers. Importantly, the increased rejection of unequal splits was not driven by a general rise in generosity but by enhanced sensitivity to social norm violations. This suggests that elevated cortical dopamine sharpens fairness-related valuations rather than prosocial tendencies per se.

A more complex bargaining paradigm [20], which incorporates elements of the Dictator and Ultimatum Games, further tested whether dopamine modulates norm-sensitive self-interest. In this paradigm, participants played the role of the proposer, distributing money to recipients across two conditions: a reward condition, where recipients had no opportunity to respond (as in the Dictator Game), and a punishment condition, where recipients could reduce the proposer’s earnings at a personal cost. The authors found that administration of the dopaminergic precursor L-DOPA led to more selfish offers—i.e., lower amounts allocated to the recipient—in the reward condition. By contrast, L-DOPA had no significant effect in the punishment condition, where unfairness could be penalised. These findings suggest that dopamine promotes self-interested behaviour when there are no social consequences, but this effect is constrained when norm violations carry the risk of punishment. This behavioural effect stands in contrast to patterns observed in studies of endogenous dopamine signalling during passive reward tasks. In two PET studies, the Zald group [21,22] found that striatal dopamine transmission was suppressed when participants passively observed unpredictable monetary outcomes, compared to conditions with predictable rewards. Given that the task required no decisions or movements, the authors suggested that dopaminergic suppression under uncertainty may be specific to contexts in which individuals lack control [23], in contrast to studies involving active engagement, which typically show increased dopamine signalling [24,25]. While the studies above emphasise dopamine’s role in modulating behaviour in relation to social agency and external controllability, additional evidence suggests that dopaminergic tone also shapes internally driven cognitive processes—such as belief formation and executive control—that influence financial decision-making. For example, Sharot et al., [26] showed that L-DOPA administration impaired belief updating in response to negative information across various life-outcome predictions, including financial risks. Participants maintained a stronger optimism bias under L-DOPA, updating their beliefs more readily in response to desirable outcomes. This asymmetric integration of feedback suggests that dopamine may bias internal representations of risk, promoting favourable expectations even in the face of adverse evidence.

A similar disruption in adaptive decision-making under reward-predictive contexts was observed in a study by Aarts et al. [27], who used PET imaging to measure baseline dopamine synthesis capacity in the striatum. In a modified Stroop task, participants were cued to expect either a high or low monetary reward and were then either informed or not informed about the upcoming trial’s difficulty. Those with higher dopamine synthesis capacity in the left caudate nucleus performed worse when anticipating a high reward without information about task difficulty. In other words, the promise of a bonus impaired cognitive control when strategic preparation was not possible. This finding suggests that elevated dopaminergic tone can interfere with goal-directed performance when motivational salience is heightened but predictive structure is lacking.

A similar theme emerges in a study by Cho et al. [28], which demonstrated that rTMS stimulation of the medial prefrontal cortex (MePFC) diminished delay discounting and increased striatal dopamine release. This finding suggests that prefrontal modulation of dopaminergic tone can enhance future-oriented valuation. Like in the Aarts et al. study, these results demonstrate that dopaminergic tone, whether elevated through endogenous synthesis or cortical excitation, shapes goal-directed behaviour under conditions where the timing or structure of rewards is uncertain. Extending this evidence, Wagner et al. [29] administered the D2 receptor antagonist haloperidol to examine its impact on intertemporal financial choices. Using drift diffusion modelling, the authors reported that haloperidol reduced impulsive choices and shortened nondecision times. These behavioural effects were interpreted as an indication of increased striatal dopamine release via presynaptic D2 autoreceptor blockade.

Together, these studies highlight that dopamine’s influence is not uniformly facilitative. It can support adaptive behaviour when actions are clearly linked to outcomes, but in less structured environments—whether due to ambiguous feedback or unpredictable task demands—dopamine may impair updating, focus, or behavioural control. This context-dependence reinforces the need to consider both task structure and agentic engagement when interpreting dopaminergic contributions to economic decision-making (see Table 1 for a summary of the studies reviewed in this section).

### 2.2. Receptor Subtype Contributions (D1, D2, D3, D4)

Whereas Section 2.1 focused on studies that manipulate overall dopamine levels or synthesis capacity, this section turns to evidence targeting specific dopamine receptor subtypes to clarify their distinct contributions to economic behaviour—most notably the D2 receptor, which remains the most extensively studied.

A PET study by Green et al. [30] explored how dopamine D2 receptor availability influences decisions in a risky investment task. Participants chose between a safe asset (bond) and a volatile one (stock), whose value could rise or fall. Lower D2 receptor binding in the amygdala was associated with choice inflexibility—participants persisted with suboptimal stock choices—while higher D2 availability predicted more adaptive switching. This finding implicates D2-mediated signalling in tracking risk–reward contingencies and adjusting preferences accordingly.

In a related pharmacological study, Arrondo et al. [31] administered the D2 receptor antagonist metoclopramide and found that it altered the trade-off between probability and delay in reward valuation. Participants under placebo showed typical encoding of subjective value, integrating both delay and probability. However, blocking D2 receptors increased participants’ willingness to wait longer for rewards with higher probabilities, suggesting a shift toward greater sensitivity to outcome certainty over immediacy. Neuroimaging revealed corresponding reductions in frontomedian and postcentral activation, indicating disrupted integration of temporal and probabilistic information.

Building on these results, van Eimeren et al. [32] used the D2 agonist bromocriptine to examine how enhanced D2 signalling modulates value-based learning. In an economic decision-making task with a reinforcement learning component (i.e., shifting reward contingencies), participants receiving bromocriptine exhibited greater cognitive flexibility, adjusting their choices more rapidly as payoff structures changed. These effects were linked to prefrontal D2 receptor stimulation and suggest that D2 activity supports dynamic updating of economic preferences.

Dopamine’s role in modulating reward sensitivity was further demonstrated in a study by Abler et al. [33], who administered olanzapine—an antagonist of D1, D2, and D4 receptors—and observed dampened BOLD responses in the ventral striatum during a monetary incentive task. This reduction in striatal activation between high- and no-reward conditions was mirrored behaviourally in slower reaction times, indicating that broad receptor blockade attenuates reward salience.

More fine-grained evidence comes from foraging-based paradigms. Heron et al. [34], administered the D2 receptor agonist cabergoline while participants engaged in a patch-leaving task designed around the Marginal Value Theorem. In this task, optimal performance required weighing the foreground reward rate (i.e., patch yield) against the background reward rate of the environment. Cabergoline administration led participants to leave low-yield patches prematurely, especially in poor environments, suggesting that overstimulation of D2 receptors inflated the perceived opportunity cost of staying. This disruption in cost–benefit computation aligns with the hypothesis that D2 activity regulates sensitivity to contextual reward structures.

D2 receptor involvement also extends to the valuation of non-monetary outcomes. Norbury et al. [35] employed a novel task in which participants chose between monetary gain and the opportunity to receive mild electric stimulation. High sensation-seekers were more likely to sacrifice money for stimulation, but this behaviour was attenuated under haloperidol, a D2 antagonist. Notably, the effect was trait-specific—present only in high sensation-seekers—suggesting that D2 signalling contributes to the subjective valuation of salient or intense sensory experiences.

Finally, Kohno et al. [36] combined PET and fMRI to examine how striatal D2/D3 receptor availability relates to behaviour on the Balloon Analogue Risk Task. Higher D2/D3 receptor binding was associated with more cautious behaviour and reduced prefrontal sensitivity to escalating risk, leading to lower earnings. This study illustrates how stable individual differences in dopamine receptor availability shape risk-taking propensities and neural responses to uncertainty.

Together, these studies highlight that D2 receptor signalling plays a central role in modulating individual differences in economic behaviour—affecting flexibility, valuation, and risk processing. While some studies implicate additional receptor subtypes (D1, D3, D4), the majority of evidence converges on the D2 receptor as a key mechanistic substrate for dopaminergic influences on financial and reward-related decision-making (see Table 2 for a summary of the studies reviewed in this section).

### 2.3. Neural Circuitry and Regional Activity

This section focuses on how dopaminergic activity within broader neural circuits—particularly corticostriatal and limbic pathways—influences economic decision-making. While Section 2.1 dealt with pharmacological or PET-based manipulations of dopamine tone, and Section 2.2 examined receptor subtype contributions, the present section shifts attention to system-level evidence derived from neuroimaging and theoretical models. These studies explore how dopamine supports reward learning, biases behaviour under affective and motivational states, and links distributed neural systems involved in valuation, memory, and risk evaluation. Though not all studies directly manipulate dopamine, their findings inform how dopaminergic signalling integrates within network-level processes that shape adaptive—and sometimes irrational—financial behaviour.

A foundational theoretical account by Bamford and Bamford [37] proposes that different striatal circuits support both rational and irrational choices. Corticostriatal loops, richly innervated by dopaminergic neurons, facilitate goal-directed learning by integrating action-outcome contingencies and updating value representations. In contrast, thalamostriatal circuits contribute to habit formation and automated behavioural responses, which can be economically maladaptive when rewards are not fully evaluated. This neural architecture may help explain why individuals sometimes exhibit ‘momentum-based’ trading behaviours—buying as prices rise and selling as they fall—patterns that ignore expected value computations and mirror early algorithmic trading rules [38]. Although this account is conceptual, it situates dopamine as a modulator of both flexible and inflexible economic responding, depending on circuit engagement.

Expanding on this system-level view, empirical studies have linked activation in specific dopaminergic hubs to biased risk-taking. Knutson et al. [39], using fMRI, found that the anticipation of viewing erotic stimuli enhanced activity in the nucleus accumbens (NAcc), a dopamine-rich region, and increased participants’ likelihood of choosing high-risk financial options. This suggests that affectively charged anticipatory states can recruit mesolimbic pathways to bias economic preferences toward risk, potentially overriding rational evaluations. A closely related finding by Kuhnen and Knutson [40] showed that increased NAcc activity predicted both risky choices and risk-seeking errors, whereas activity in the anterior insula—a region linked to negative affect and loss anticipation—predicted risk-averse decisions. These results demonstrate a neural double dissociation in which dopaminergic reward signals promote risk-taking while insular responses may act as a brake on such tendencies.

Although many studies link dopamine to reward valuation and action selection, Adcock et al. [41] highlight its role in learning and memory, showing that motivational salience can enhance encoding. In a monetary incentive delay task, they found that high-reward cues triggered anticipatory activity in the ventral tegmental area (VTA), NAcc, and hippocampus, but only for stimuli that were later remembered. Importantly, VTA-hippocampal functional connectivity predicted subsequent memory accuracy, suggesting that dopaminergic signalling may facilitate the transfer of motivational salience into long-term memory formation. This aligns with broader accounts in which dopamine supports not just immediate decision-making, but the construction of internal models that guide future behaviour.

Building on this idea, Treadway et al. [42] used PET to understand how individual variability in dopamine reactivity across brain regions shapes motivational preferences in effort-based decision-making. The authors found that greater dopamine release in the left striatum and ventromedial prefrontal cortex was associated with a higher willingness to expend effort for uncertain but larger rewards, suggesting enhanced reward sensitivity under dopaminergic facilitation. On the other hand, in the insula, increased dopamine responses implicated in cost encoding—were linked to reduced effort investment, suggesting a neurochemical mechanism for cost–benefit weighting. These findings reinforce the view that dopamine operates across distributed neural circuits to shape how individuals evaluate and act on economic opportunities, particularly under uncertainty and effort constraints.

Together, these studies illustrate that dopamine’s influence on economic behaviour is not restricted to valuation at the point of choice. Instead, it operates across distributed neural systems, shaping learning, anticipation, and affective engagement in ways that can bias financial behaviour toward or away from optimality. Whether through goal-directed corticostriatal control, emotionally driven mesolimbic activation, or memory-enhancing VTA–hippocampal coupling, dopamine dynamically modulates how individuals process and respond to value-related information over time (see Table 3 for a summary of the studies reviewed in this section).

### 2.4. Genetic Polymorphisms Affecting Dopamine Signalling

This section reviews studies examining how individual differences in dopaminergic genes influence economic behaviour, particularly in temporal discounting, risk-taking, norm sensitivity, and adaptive learning. In contrast to pharmacological or imaging approaches, which capture dynamic or acute changes in dopamine function, genetic studies offer insight into more stable predispositions shaped by variations in dopamine-related genes, such as DRD4, DRD2, COMT, ANKK1, DAT1, and MAOA. These polymorphisms influence receptor availability, dopamine clearance rates, and regional dopamine tone, thereby shaping trait-like tendencies in valuation, impulsivity, and social preferences.

One prominent behavioural domain examined in this context is temporal discounting, which serves as a proxy for impulsivity and future-oriented decision-making. Carpenter et al. [43] used a dual-block lottery task to compare preferences for immediate versus delayed rewards. Individuals carrying the DRD4 7-repeat allele (7R+), which is associated with reduced D4 receptor sensitivity, displayed a distinctive “anti-hyperbolic” pattern—discounting delayed outcomes more steeply when both options were in the future. This suggests that impaired dopaminergic modulation of cognitive control may undermine future-oriented valuation, particularly when the choice lacks immediate salience. Similarly, McGeary [44] found that polymorphisms in COMT and ANKK1 (ankyrin repeat and kinase domain containing 1) predicted greater preference for immediate rewards, likely reflecting reduced prefrontal dopamine availability (due to more active COMT catabolism) and lower striatal D2 receptor density, respectively. These dopaminergic inefficiencies may bias decision-making toward short-term gains by weakening regulatory control over impulsive tendencies.

Beyond impulsivity and delay-based valuation, dopaminergic gene variants have also been implicated in social economic behaviour, particularly in contexts involving fairness, norm adherence, and social learning. These traits are frequently studied using paradigms such as the Ultimatum Game or Dictator Game, which isolate different facets of distributive justice and reciprocity. While temporal discounting tasks probe individual preferences in isolation, these social paradigms capture how dopamine may shape interpersonal expectations and norm sensitivity under conditions of reward division and strategic interaction.

Jin et al. [45] applied computational modelling and fMRI in a twin sample, showing that norm learning rates—but not fairness valuation—were linked to DRD2 variants and attenuated prediction error signals in the medial frontal cortex. This suggests that D2 receptor signalling may specifically support the flexible updating of social expectations via dopaminergic modulation of medial frontal learning circuits. Other studies linked DRD4 to responder behaviour, with 4-repeat homozygotes requesting higher minimum acceptable offers [46,47], suggesting that greater D4 receptor efficiency may enhance perceived entitlement. Additionally, DRD2 haplotypes were associated with proposer generosity, possibly reflecting dopaminergic modulation of social conformity. Neural sensitivity to norm violations was further supported by EEG evidence: DRD4 7R+ carriers showed enhanced feedback-related negativity (FRN) in a Dictator Game, reflecting heightened expectancy violation responses [48]. This pattern implies that reduced D4 receptor function amplifies prediction error signals when social norms are breached, potentially increasing the salience of unfair treatment even in non-reciprocal contexts.

Whereas the previous studies highlight dopamine’s role in social norm sensitivity and fairness-related decision-making, other lines of research have focused on adaptive learning, feedback monitoring, and risk-based financial behaviour. These areas focus on how individuals adjust their strategies based on dynamic environmental feedback, engaging distinct dopaminergic mechanisms that support prediction error signalling, behavioural flexibility, and valuation of uncertain outcomes.

In a competitive financial patent race task, strategic adjustments were analysed using computational modelling. Variability in COMT predicted differences in belief learning (δ), likely reflecting dopaminergic modulation of prefrontal circuits involved in goal-updating. In contrast, variations in DRD2 and DAT1 were associated with the learning rate (ρ), suggesting that striatal dopamine supports temporal flexibility in updating reward contingencies [49].

Similar insights come from feedback-monitoring paradigms. Heitland et al. [50] reported that carriers of the DAT1 9-repeat allele showed stronger feedback-related negativity (FRN) following losses, consistent with enhanced dopaminergic prediction error signalling in the striatum [51]. Meanwhile, COMT met/met homozygotes displayed larger P3 amplitudes after both gains and losses, implicating prefrontal dopamine in conscious rule-based adaptation and salience detection.

Dopaminergic genes have also been linked to individual differences in financial risk-taking. Several studies found that DRD4 7R+ carriers—particularly men—exhibited higher risk tolerance across investment and gambling tasks [52,53,54]. These findings suggest that reduced D4 receptor efficiency may lower aversive responses to uncertainty, thereby facilitating approach behaviour under risk. However, replication has been inconsistent: other studies using Holt–Laury tasks or self-report measures failed to find significant differences between 7R+ and 7R− individuals [55].

Finally, monoamine oxidase A (MAOA) polymorphisms may offer a more robust explanation for risk-related variability. In one study [56], MAOA-L carriers were more likely to select risky financial gambles, but this pattern reflected selective responsiveness to advantageous risks—not impulsivity. Given MAOA’s role in catabolising multiple monoamines, including dopamine, this result highlights the broader neurochemical architecture involved in risk assessment.

Together, these findings underscore the domain-specific roles of dopaminergic polymorphisms across valuation, feedback, and learning systems, while also revealing sex-, context-, and task-dependent effects that challenge unitary models of dopamine and decision-making (see Table 4 for a summary of the studies reviewed in this section).

### 2.5. Stable Trait-Level Influences

This final section examines how relatively stable dopaminergic characteristics—such as genetic polymorphisms and structural brain features—are associated with long-term economic preferences and behavioural strategies. Although some findings are cross-sectional, they suggest that trait-level dopaminergic mechanisms, including variation in catecholamine-related genes or connectivity in dopamine-rich brain regions, may contribute to the emergence and maintenance of distinct economic decision-making styles—for example, among seasoned investors or individuals under chronic financial stress.

One study investigated structural and genetic differences between senior and junior investors, finding that senior investors exhibited greater grey matter volume and enhanced structural connectivity in dopamine-rich regions [57]. Genetic analyses revealed differences in catecholamine-related genes (SLC6A3, TH, SLC18A2), suggesting that long-term investment experience may reflect or reinforce dopaminergic traits linked to reward sensitivity and stress resilience. Extending these findings, another study showed that variation in the DAT1 gene moderated the relationship between financial stress and intimate partner violence [58], implying that dopamine transporter efficiency may broadly shape behavioural responses to economic pressure by regulating emotional reactivity. Supporting this broader view, a separate investigation found that Wall Street traders with DRD4 and COMT variants linked to moderate synaptic dopamine levels had longer trading careers [59], consistent with the idea that optimal dopaminergic tone may promote sustained performance in high-stakes financial environments (see Table 5 for a summary of the studies reviewed in this section).

## 3. Integrative Synthesis

The evidence reviewed across multiple levels of analysis shows that dopamine plays a mechanistic and multifaceted role in economic decision-making, shaping valuation, behavioural flexibility, and sensitivity to uncertainty. Section 2.1 demonstrated that manipulating dopaminergic tone can modulate economic behaviour in a context-sensitive manner. Elevated dopamine—via L-DOPA, tolcapone, or rTMS—can either enhance norm sensitivity (e.g., in fairness paradigms) or promote self-interest and optimism bias when social or structural constraints are absent. Crucially, excessive dopamine impairs belief updating and cognitive control under ambiguous reward contingencies, suggesting a non-linear relationship between dopamine and adaptive behaviour. These findings underscore dopamine’s dual role: facilitating goal-directed valuation when contingencies are clear, but distorting belief updating and decision strategies when outcomes are uncertain or feedback is ambiguous, leading to overly optimistic or maladaptive internal representations.

Section 2.2 studies across investment, learning, and foraging tasks show that D2 receptor signalling is a key modulator of economic behaviour. Higher D2 availability enhances flexible adaptation to shifting contingencies, while antagonism alters valuation strategies, increasing delay tolerance while also impairing probabilistic integration. D2 stimulation also inflates perceived opportunity costs and modulates the evaluation of salient sensory outcomes, but only among high sensation-seekers, suggesting trait-contingent effects. Though the role of D1, D3, and D4 receptors remain largely understudied, converging data identify D2-mediated prefrontal and striatal mechanisms as central to risk sensitivity, reward tracking, and behavioural updating.

Studies from Section 2.3 show that dopamine modulates economic behaviour via distributed neural circuits involved in reward, emotion, memory, and effort valuation. Neuroimaging investigations suggest an association between mesolimbic activity (e.g., nucleus accumbens, VTA) and risk-seeking under affective arousal, in addition to enhanced memory for reward-predictive cues. Dopaminergic signalling also affects effort-based decision-making via cost–benefit encoding in the insula and striatum. Theoretical models further suggest that distinct striatal loops underlie goal-directed vs. habitual financial choices, positioning dopamine as a regulator of both adaptive and maladaptive strategies depending on circuit engagement.

Studies in Section 2.4 show that dopamine-related genetic polymorphisms modulate individual differences in economic preferences, specifically in temporal discounting, norm sensitivity, and adaptive learning. Variants in DRD4, COMT, and ANKK1 are linked to impulsive choice and future devaluation, while DRD2 and DAT1 modulate social learning and feedback updating via striatal and prefrontal pathways. Electrophysiological (i.e., EEG) and neuroimaging (i.e., fMRI) studies show that these effects are mediated by dopaminergic prediction error signalling and trait-level responsiveness to uncertainty. While some associations (e.g., DRD4 and risk-taking) remain inconsistent, these findings highlight dopamine’s role in domain-specific, genotype-dependent economic behaviour.

Section 2.5 highlights preliminary evidence that stable dopaminergic traits—such as genetic variants in catecholamine-related genes and structural connectivity in dopamine-rich brain regions—may shape long-term economic strategies. Findings from investors and traders suggest that optimal dopamine function supports stress resilience and sustained decision performance, though is important to stress that evidence remains limited and cross-sectional.

Future research would benefit from the integration of pharmacological or neuromodulatory interventions with computational modelling and multimodal imaging to clarify causal mechanisms linking dopamine to financial decision-making. Longitudinal studies are also needed to understand how trait-level dopaminergic factors interact with dynamic state changes during real-world, high-stakes economic choices.

## Figures and Tables

**Table 1:** Summary of key studies on Dopamine Release and Pharmacological Modulation.

Study	Methodology	Targeted Dopaminergic Feature	Economic Paradigm	Key Finding
[18]	Deep Brain Stimulation	Dopamine release in the Substantia Nigra	Ultimatum Game	Greater dopamine release during socially motivated rejections.
[19]	Tolcapone administration and computational modelling	Prefrontal dopamine tone via COMT inhibition	Dictator Game	Tolcapone increased rejection sensitivity to unfair offers.
[20]	L-Dopa administration	Acute increase in dopaminergic tone	Bargaining paradigm	Dopamine increases selfishness absent social punishment risk
[21]	PET imaging	Dopamine transmission in medial caudate and putamen	Card selection task	Unpredictable passive rewards suppressed dopamine in putamen
[22]	PET imaging	Dopamine transmission in striatum (putamen focus)	Passive Roulette Game	Unpredictable rewards altered dopamine across striatal regions
[26]	L-Dopa administration	Acute increase in dopaminergic tone	Belief updating under risk	L-DOPA increased optimism bias in risk beliefs
[27]	PET imaging	Dopamine synthesis capacity in left caudate	Reward-modulated cognitive control (modified Stroop task)	High dopamine impaired control under reward uncertainty
[28]	rTMS stimulation + [^11^C]raclopride PET imaging	Dopamine transmission in striatum (via prefrontal modulation)	Delay discounting (intertemporal choice task)	Prefrontal stimulation enhanced patience, increased striatal dopamine
[29]	Haloperidol administration + drift diffusion modelling	D2 autoreceptor blockade (increased striatal dopamine release)	Delay discounting (intertemporal choice task)	Haloperidol reduced impulsivity, increased dopamine tone

**Table 2:** Summary of key studies on receptor subtype contributions.

Study	Methodology	Targeted Dopaminergic Feature	Economic Paradigm	Key Finding
[30]	PET imaging	D2 receptor binding in amygdala	Risky investment task (stock vs. bond choices)	Lower D2 binding linked to inflexible risk-taking
[31]	Metoclopramide + fMRI	D2 receptor blockade	Intertemporal and probabilistic reward valuation	D2 blockade increased preference for high-certainty delays
[32]	Bromocriptine administration	D2 receptor stimulation	Reinforcement learning with shifting reward contingencies	Bromocriptine enhanced flexibility in reward-based updating
[33]	Olanzapine administration + fMRI	D1/D2/D4 receptor antagonism	Monetary incentive task	Receptor blockade reduced reward salience, slowed response
[34]	Cabergoline administration	D2 receptor agonism	Patch-leaving task	D2 stimulation inflated opportunity cost, impaired foraging decisions
[35]	Haloperidol administration	D2 receptor antagonism	Monetary vs. aversive stimulation choice task	D2 blockade reduced sensation-seeking valuation bias
[36]	PET and fMRI	Striatal D2/D3 receptor availability	Balloon Analogue Risk Task (BART)	Higher D2/D3 binding linked to cautious risk-taking

**Table 3:** Summary of key studies on neural circuitry and regional activity.

Study	Methodology	Targeted Dopaminergic Feature	Economic Paradigm	Key Finding
[39]	fMRI	Nucleus accumbens (NAcc) activation	Risky financial choice task	Affective cues increased NAcc activity and risk-taking
[40]	fMRI	Nucleus accumbens (NAcc) activation	Risky investment task	NAcc predicts risk-taking; insula predicts caution
[41]	fMRI	VTA–hippocampus functional connectivity	Monetary Incentive Delay task	Reward anticipation enhances memory via VTA-hippocampus link
[42]	PET imaging	Dopamine release in striatum, vmPFC, and insula	Effort-based decision-making task	Striatal/vmPFC dopamine promotes effort for reward

**Table 4:** Summary of key studies on genetic polymorphisms affecting dopamine signalling.

Study	Methodology	Targeted Dopaminergic Feature	Economic Paradigm	Key Finding
[43]	Genetic association study	DRD4 7-repeat allele (D4 receptor sensitivity)	Temporal discounting (dual-block lottery task)	DRD4 variant impairs delayed reward valuation
[44]	Genetic association study	COMT and ANKK1	Temporal discounting/reward preference	COMT/ANKK1 linked to impulsive reward bias
[45]	fMRI with computational modelling in twin sample	DRD2 variant effects	Social norm learning/fairness-based decision-making	DRD2 linked to norm updating, not fairness value
[46]	Genetic association study	DRD4 exon III, DRD2 haplotype	Ultimatum Game	DRD4 and DRD2 variants shape fairness preferences
[47]	Genetic association study	DRD4 exon III polymorphism	Ultimatum Game	DRD4 genotype linked to fairness preferences
[48]	Genetic association + EEG (FRN)	DRD4 exon III (7-repeat allele)	Dictator Game	7R+ carriers show stronger norm violation signals
[49]	Genetic association + computational modelling	COMT, DRD2, DAT1 polymorphisms	Financial patent race task	COMT, DRD2, DAT1 shape distinct learning processes
[50]	Genetic association study with EEG	DAT1, COMT polymorphisms	Feedback-monitoring task	DAT1, COMT variants modulate feedback monitoring signals
[52]	Genetic association study	DRD4 7-repeat allele (7R+)	Financial risk-taking task with monetary payoff	DRD4 7R+ men showed greater risk tolerance
[53]	Genetic association study	DRD4 7-repeat allele (7R+)	Bridge-based strategic risk task and financial gamble	7R+ men took more strategic and financial risks
[54]	Genetic association study	DRD4 7-repeat allele (7R+)	Investment risk-taking task	7R+ carriers took significantly greater financial risks
[55]	Genetic association study	DRD4 7-repeat allele (7R+)	Holt–Laury task; investment motivation questionnaire	No risk-taking difference between 7R+ and 7R−
[56]	Genetic association study	MAOA	Risky financial gambles	MAOA-L carriers preferred advantageous financial risk choices

**Table 5:** Summary of key studies on stable trait-level influences.

Study	Methodology	Targeted Dopaminergic Feature	Economic Paradigm	Key Finding
[57]	Structural MRI and genetic association study	Grey matter/connectivity in dopamine-rich regions; catecholamine-related gene variants (SLC6A3, TH, SLC18A2)	Investment experience (long-term investor classification)	Senior investors showed enhanced dopamine-related brain traits
[58]	Genetic association study	DAT1, DRD2, DRD4 gene–environment interaction	Financial stress exposure	DAT1 amplified intimate partner violence under stress; DRD2 reversed
[59]	Genetic association study	DRD4 and COMT variants	Real-world occupational data (Wall Street traders)	DRD4 promoter and COMT alleles linked to balanced synaptic dopamine levels predicted longer trading careers

## Data Availability

No new data were created or analysed in this study. Data sharing is not applicable to this article.
